# The influence of social signals on the self-experience of pain: A neuroimaging review

**DOI:** 10.3389/fneur.2022.856874

**Published:** 2022-08-26

**Authors:** Gil Sharvit, Petra Schweinhardt

**Affiliations:** ^1^Department of Chiropractic Medicine, Integrative Spinal Research, Balgrist University Hospital, University of Zurich (UZH), Zurich, Switzerland; ^2^Neuroscience Center Zurich (ZNZ), University of Zurich (UZH), Zurich, Switzerland

**Keywords:** pain, social, neuroimaging, social effects, pain modulation

## Abstract

Researchers in cognitive neuroscience have investigated extensively how psychological factors shape the processing and perception of pain using behavioral, physiological, and neuroimaging methods. However, social influences of pain, an essential part of biopsychosocial pain models, have received relatively little attention. This is particularly true for the neurobiological mechanisms underlying social modulations on pain. Therefore, this review discusses the findings of recent neuroimaging studies measuring the effects of social manipulations on pain perception (e.g., verbal and non-verbal social signals, social interaction style, conformity, social support, and sociocultural mediators). Finally, a schematic summary of the different social modulatory themes is presented.

## Introduction

In 2020, the International Association for the Study of Pain (IASP) revised the original definition of pain from 1979 (1), which now reads: “An unpleasant sensory and emotional experience associated with, or resembling that associated with, actual or potential tissue damage”. Notably, the updated definition includes the following integral note: “Pain is always a personal experience that is influenced to varying degrees by biological, psychological, and social factors”. Thus, the IASP's new definition recognizes that pain can also be influenced by social factors, which was not contained in the original definition. This recognition stems from accumulating evidence for biopsychosocial models of pain, which illustrate that different social contexts can influence an individual's experience of pain.

Over the centuries, different pain models have emerged to conceptualize the root causes of pain to offer better treatment for those who suffer. One of the significant milestones in this progress was made by Engel (2), who proposed a new conceptualization of illness that was different from existing biomedical frameworks, which viewed illness and its symptoms, such as pain, as an integration of social, psychological, and behavioral influences. Since Engle's initial model, other biopsychosocial models, variants of the initial model, have been theorized in the pain field (3–6).

With the advancements in noninvasive experimentation and measurement techniques in humans (e.g., neuroimaging), substantial knowledge on how pain is experienced, processed in the brain, and modulated by different biological and psychological or cognitive factors has been accumulated [see Schweinhardt and Bushnell (7), Villemure and Bushnell (8), and Tracey and Mantyh (9) for detailed reviews]. Cognitive factors include attention (8), cognitive appraisals (10), and expectations (11).

In contrast to the research on psychological and biological influences on pain, modulation by social factors receive less attention. This conclusion is based on three primary assessments: first, the original IASP definition of pain did not include any aspect of potential social influence on pain. This recognition only came with the new, revised IASP definition within its notes (1). Second, a simple search in PubMed using the word combinations of “pain” with “psychological factor,” “biological factor,” or “social factor” reveals 84,134 hits, 56,953 hits, and 13,016 hits, respectively. Furthermore, looking at the first date of published studies, the first combination starts in 1912, whereas the combination of “pain” with “social” shows the studies starting only from 1964. Third, the first conceptualization of biopsychosocial models of pain was only published in 1977 by Engel (2). Consequentially, there are also fewer review articles on pain modulation that include social factors, even though a biopsychosocial conceptualization of pain is today's gold standard (5, 6, 12).

At the time of this review, the most recent review examining the influence of a range of social factors on pain was published by Krahé et al. (13) and included only three neuroimaging studies. More recent neuroimaging-focused reviews on social pain exist; however, they either focus on one specific social factor (14–16) or are not solely focused on pain modulation (17).

Therefore, there is a need to close the gap in the neuroimaging literature on pain modulations by social factors. This review provides an updated view on the topic by including studies up to March 2021. In addition, an essential aim of this review is to synthesize the findings of the individual studies across different social factors regarding the brain structures involved in the social modulation of pain. First, it is predicted that social manipulations modulate pain processing in pain-related brain areas (e.g., insular cortex, cingulate cortex) (9, 18, 19). Second, it is hypothesized that brain regions mediating such modulations are consistently recruited across several social themes. In particular, the prefrontal cortex is expected to mediate social context effects on pain because it has been previously shown that the prefrontal regions (e.g., the dorsolateral prefrontal cortex [DLPFC]) modulate pain expectancy effects on subjective ratings (11, 20, 21).

In this review, at first, a search using combinations of general keywords (“social,” “neuroimaging,” and “pain”) was applied to extract a large number of studies. Then, more specific keywords were used in later search iterations. These specific keywords were based on previous reviews of social modulations on pain—both behavioral and neuroimaging (13–16). Accordingly, relevant keywords that were mentioned/investigated previously with this topic, such as “attachment”/“social attachment” (22–24), were included.

**Figure 1:**
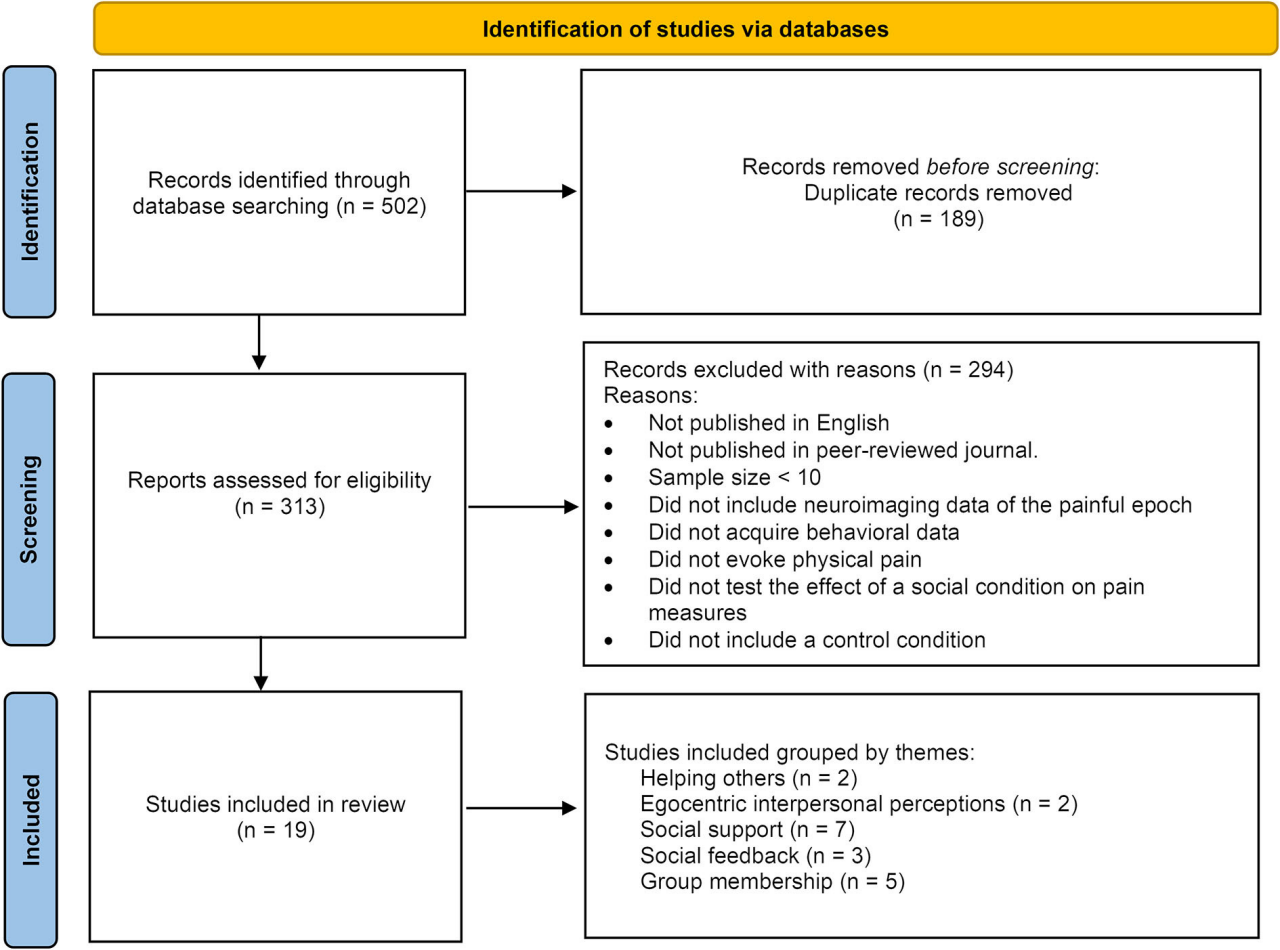
Summary of the study selection pipeline.

After applying both general and specific search queries across all databases, the data were filtered for relevance, inclusion, and exclusion criteria (as specified in the Methods section), and duplicates were removed from the total of all the search iterations (as summarized in Figure 1, and fully detailed in the Supplementary material section).

Next, the remaining studies were clustered into logical thematic classifications. The naming of the clusters was chosen based on either a known classification used in the literature (e.g., “Social Support,” “Group Membership,” and “Social Feedback”) or by choosing a name based on social manipulation features that were common across the different manipulations (e.g., “Egocentric Interpersonal Perceptions,” and “Helping others”). After presenting the results, an overall synthesis of the findings within and across all the resulting themes is provided in the Discussion.

## Methods

### Search strategy

On March 15, 2021, a search of the online databases PubMed, Google Scholar, and Scopus was conducted using different combinations of some (but not all) of the following keywords with “pain”: “interpersonal,” “attachment,” “social context,” “social interaction,” “social support,” “social presence,” “social modulation,” “social media,” “social manipulation,” “social intervention,” “social behavior,” “peer support,” “social signals,” “social cues,” “social communication” and “communication”. These topical combinations were searched in conjunction with words describing neuroimaging approaches such as “neuroimaging,” “imaging,” “fMRI,” “functional magnetic resonance imaging,” “PET,” “positron emission tomography,” “EEG,” “electroencephalogram,” “EP,” “evoked potentials,” “MEG,” and “magnetoencephalography”.

For full details on the different searches conducted (consisting of all the keywords used in each database and search iteration), please see the Supplementary methods section.

The primary search included these combinations using the title and abstract of the publications. Next, the search was repeated using keywords integrated within each database, such as the medical subject headings (MeSH) terms in PubMed and index terms (controlled vocabulary terms assigned to the document) in Scopus.

In addition, reference lists of relevant articles were searched. No restrictions regarding the publication date were applied.

### Study selection—inclusion/exclusion criteria

PubMed, Google Scholar, and Scopus were used for the search in a step-by-step approach (removing duplicates, applying exclusion criteria, and manually reviewing and rechecking the final extract), as seen in Figure 1.

The core aim of this review is to present and discuss the main findings of studies investigating how social signals can alter the processing and the perception of pain. For this purpose, three guiding rules were set in advance regarding the definition of social manipulation, the pain induction method, and the pain delivery target. A social manipulation was considered as one in which the main test/condition includes interaction between a participant and others—either in real time (e.g., having another person present with the participant) or in offline mode (e.g., observing evaluations of other people). In comparison, psychological manipulation does not include any form of social interaction (e.g., anxiety induction, fear conditioning, stimulus expectancy, learning task, etc.). Purposefully, only studies in which pain was administered to the individual by physical means (e.g., thermal pain, electrical pain, pressure pain) and not by non-physical means (e.g., inferred/believed pain) were included. The rationale here was to focus on studies that investigate modulations occurring on a type of pain in which the biological mechanisms of the induced pain i) are mostly known, ii) easily replicated and controlled, and iii) allows for better dissociation of any external modulations (e.g., social manipulations) on one type of pain, and iv) avoid mixing different types of pain in a single review.

This yielded two thematic concepts that were left out from this review: studies focusing on empathy for pain (“others' pain”) without examining how empathy affects individuals experiencing pain and those using social inclusion/exclusion tasks to induce “social pain”—a concept which is still debated regarding the degree of shared features with physical pain (25–28). Please note that studies on empathy for pain were included in this review only when empathy for pain was used as a social manipulation of which the effect on individuals' perception or processing of physical pain stimuli was tested. Similarly, studies using social inclusion/exclusion tasks were included if they tested the effect of the social inclusion/exclusion perception or processing of physical pain stimuli.

Following the thematic restrictions, the following inclusion/exclusion criteria were applied to the search results:

#### Inclusion criteria

The inclusion studies were neuroimaging studies that

1. Delivered experimental painful stimuli to healthy participants or clinical pain patients by using, e.g., thermal stimuli (e.g., by a thermode, laser, or a cold-water bath), electrical stimuli, or mechanical stimuli (e.g., by an inflatable cuff).
2. Reported behavioral and/or physiological data.
3. Reported the main effect of a social manipulation on neural activation in response to painful stimuli (i.e., not only reporting effects of external modulators (e.g., questionnaires data) on neural activation).
4. Published in English in a peer-reviewed scientific journal.
5. Conducted controlled experiments on human participants older than 18 years old.

#### Exclusion criteria

The exculsion criteria were as follows:

1. Clinical painful procedures (e.g., wisdom teeth extraction);
2. Neuroimaging studies that tested a very small sample (< 10 participants);
3. Neuroimaging studies on empathy for pain (“others' pain”); and
4. Those using social inclusion/exclusion tasks to induce “social pain”—unless the effects of the observation of “others' pain” or social inclusion/exclusion tasks were used as a manipulation to test for effects on pain perception during pain delivery.

### Validation of coordinates

All the studies included in this review reported the fMRI coordinates in the Montreal Neurological Institute (MNI) space. To ensure that the studies assigned the reported activation clusters to the correct brain region, the studies' peak-voxel coordinates were cross-checked for each cluster by entering the reported coordinates into the Automated Anatomic Labeling (aal3v1) brain atlas, accessed by the WFU PickAtlas SPM toolbox (http://fmri.wfubmc.edu/software/pickatlas). In addition, the reported coordinates were double-checked with Neurosynth (http://neurosynth.org) (24) and NeuroQuery (https://neuroquery.org/) (25), which are automated meta-analytic tools that produce fMRI brain maps. NeuroQuery focuses on producing a brain map that predicts where in the brain a study on the topic of interest is likely to report observations, while Neurosynth tests the consistency of observations reported in the literature. An MNI coordinate was classified as a true positive if is located within the labeled regions by the aa3v1 brain atlas and lying within the Neurosynth or NeuroQuery meta-analytic brain region.

## Results

### Search results

A total of 502 studies were retrieved initially, and after the study selection step (applying inclusion/exclusion criteria described in the Methods section above) and duplicates removal (see Supplementary methods), the search results yielded a final selection of 19 studies for this review (Table 1). Based on the manipulation used, these studies were grouped into five thematic subjects: helping others, egocentric interpersonal perceptions, social support, social feedback, and group membership. In the following sections, the results of these studies are presented and discussed in the context of these thematic groups.

**Table 1:** Summary of results.

**Theme**	**Topic**	**Ref**.	**Group(s)**	**Main conditions**	**Main contrasts**	**Behavioral results**	Neuroimaging results*	**Modulations/Mediations**
**Helping others**	***Altruistic behavior***	Wang et al. (29)	32 HC (14M|18F)	*Altruistic decision* Donate/do not donate money to an orphan	Altruistic vs. Control (matching visual task)	↓pain ratings	↓ pain-related areas (bil INS, ACC, SI), r caudate, MFG, IPL ↑ FC: VMPFC—dACC ↑ FC: VMPFC (donation phase)—r AI	↑ perceived helpfulness ∞↓ dACC, ↓ bil INS Number of altruistic (vs. control) decisions ∞↑ VMPFC (at donation phase) ↑ VMPFC (at donation phase) ∞↑ perceived helpfulness, ↓ dACC, ↓ bil INS
	***Prosocial meaning***	López-Solà et al. (30)	29 HC(29F) + Partners (> 3 months)	*Prosocial decision* accept % of partner's pain stimuli (25%-75%) *Baseline* (without prosocial decision)	Accept-Partner-Pain vs. Control (pain without a decision)	↑positive thoughts ↓pain unpleasantness ↔ pain intensity	↓ l AI, r OFC ↑ VMPFC, r thalamus ↔ NPS	↑ partner pain acceptance ∞↓ pain ratings, ↑ positive thoughts, ↑VMPFC ↑ positive thoughts ∞↓ NPS ↓ (MCC, bil AI, SI, r LPFC, OFC) ∞↓ pain ratings, ↓ NPS ↑ VMPFC, ↓ r OFC ∞↓pain-related areas (bil AI, MCC, SI)
**Egocentric interpersonal perceptions**	***Observing others***	Valeriani et al. (31)	12 HC (7M|5F)	*Video watching* Static Hand/Needle in Hand/Q-tip on Hand/Needle in Foot/Needle in Tomato	Across all conditions	↔ pain ratings		N.A.
					Needle in Hand vs. All other conditions	↑self/other-referred pain ratings	↓ N1, P1 LEPs ↔ N2, P2 LEPs	↑ difference in self-other referred pain ratings ∞↓ N1/P1 LEPs ↑ stimulus pain ratings ∞↑ self-referred pain ratings
					Needle in Hand/Foot vs. all other conditions	↑self/other referred pain ratings Rating: Other-referred pain > Stimulus pain > Self-referred pain		N.A.
	***Patient-clinician interaction***	Ellingsen et al. (32)	17 FM Patients (17F) 17 Clinicians (5M|12F)	*Social interaction* (clinical interview, pre-task): Interaction/No interaction *Sham treatmen*t (pre-task, unobservable) *Clinician treatment decision(observable*) Electroacupuncture (EA) Treatment/No treatment	Social interaction vs. No interaction	↔pain ratings		N.A.
					EA vs. Sham EA	↔pain ratings	
					Treat vs. No Treat observed decision	↓pain ratings ↓ clinicians' vicarious pain ratings ↑ patient/clinician positive feeling	↑ VLPFC, TPJ, DLPFC, MPFC, Precuneus, IPL	↓ pain ratings ∞↓ clinicians' vicarious pain ratings, ↑ relationship quality ratings (during scanning session), ↑ clinicians' accuracy in treatment efficacy estimation ↑ patient-clinician facial mirroring (at anticipation) ∞↓ pain ratings, ↑ therapeutic alliance ratings (during the pre-scan social interaction) ↑ treatment analgesia ∞↑ r VLPFC, Precuneus, IPL, SMG
**Social support**	***Social viewing***	Younger et al. (33)	15 HC (7M|8F) + Partners (<9 months)	*Viewing task* Partner's picture/Baseline (acquaintance's picture) *Distraction task*	Partner viewing/Distraction vs. Baseline	↓pain ratings	N.A.	↓ pain ratings ∞↑ r OFC
					Partner viewing vs. Distraction/Baseline	↓pain ratings	↓ bil PI, l thalamus, r DLPFC, r SI ↑ ACC, MCC, bil OFC, l Amyg, Precuneus	↓ pain ratings ∞↑ r thalamus, bil caudate, bil Nac, r DLPFC, bil OFC, l Amyg, r STG ↓ pain ratings ∞↓ l AI, ACC, l SFG, r brainstem, l hippocampus
					Partner viewing vs. Distraction	↔pain ratings		N.A.
		Eisenberger et al. (34)	21 HC (21F) + Partners (9 months to 13 years)	*Viewing task* Partner's picture/Stranger's picture/Object's picture	Stranger vs. Object viewing	↔ pain ratings		N.A.
					Partner vs. Stranger/Object viewing	↓ pain ratings	↓ pain-related areas (dACC, bilateral AI) ↑ VMPFC, SMA	↑ VMPFC ∞↓ dACC, ↑ relationship-length, ↑ perceived support ↓ pain ratings ∞↑ VMPFC, ↓ dACC
					Stranger vs. Partner viewing	↑ pain ratings	↑ l caudate	N.A.
		Che et al. (35)	20 HC (8M|12F) + Partners (identified as being in a romantic relationship)	*Viewing task* Partner's picture/Stranger's picture *TMS on DMPFC* Pre iTBS/Post iTBS/Sham.	Partner vs. Stranger viewing (Pre l DMPFC- iTBS)	↓pain ratings ↑ perceived support		N.A.
					Partner vs. Stranger viewing (Post vs. Pre l DMPFC-iTBS)	↓pain ratings	
					Partner viewing (Post vs. Pre l DMPFC- iTBS)	↔ pain ratings ↓ perceived support	↑fronto-central gamma activity ↑fronto-occipital alpha connectivity	↑ gamma activity ∞↑ N100 amplitude
					Stranger viewing (Post vs. Pre l DMPFC-iTBS)	↑ pain ratings ↔ perceived support	↑central-parietal gamma activity ↑central-frontal & central-parietal theta connectivity	N.A.
	***Social touch***	von Mohr et al. (36)	29 HC (29F) + Partners (>1 year)	*Receiving a tactile touch from the partne*r* Affective (slow) touch / Neutral (fast) touch *locations differ from noxious stimuli	Affective (slow) vs. Neutral (fast) touch	↓pain ratings ↑ comfort, pleasantness	↓ N1, N2, P2 LEPs	↑ attachment anxiety score ∞↓ diff in pain ratings between slow & fast touch
		Kreuder et al. (37)	46 HC (30M|16F) + Partners (> 5 months)	*Social Suppor*t Handholding with Partner/Handholding with Stranger / No handholding	Across all conditions	Pain ratings: Partner < Stranger < No support	N.A.	N.A.
					Partner/Stranger support vs. No support	↓pain ratings	↓l AI	
					Partner vs. Stranger/No support	↓pain ratings	↑r MFG ↓FC: r MFG—r AI, r MFG—l Amyg	
					Partner vs. Stranger support (females only)	↓pain ratings	↑r VMPFC	
					Partner vs. No support (females only)	↓pain ratings	↑l thalamus, l caudate	
					Stranger support vs. No support (females only)	↓pain ratings	↑r VMPFC, l OFC	
		López-Solà et al. (38)	30 HC (30F) + Partners (> 3 months)	*Social Suppor*t Handholding with partner/Baseline (Holding rubber)	Partner support vs. Object (rubber squeeze ball)	↓pain ratings ↑emotional comfort	↓ pain-related areas (ACC, l AI, PAG, S1, l thalamus), frontal areas (bil DLPFC, bil OFC, bil MPFC), l Amyg ↓ NPS ↑FC: NPS with DMN, NAc, MTG, SI	↓ prefrontal brain areas (DLPFC, VLPFC, DMPFC, VMPFC, OFC), Amyg, ACC, PAG ∞ ↓ pain ratings ↑ emotional comfort ∞ ↓ pain ratings, ↑ perceived relationship quality ↓ NPS didn't correlate with ↓ pain ratings ↑ FC between NPS-SI ∞↓ pain ratings
	***Social presence***	Krahé et al. (39)	31 HC (31F) + Partners (> 1 year)	*Partner presence*/Absence *Partner focus* Participant/Stranger	Partner presence vs. Absence	↔ pain ratings	↑ P2 LEP	↑ attachment avoidance ∞↑ pain ratings, ↑ N2 and P2 LEPs ↑ attachment anxiety ∞↓ latency of N1 and N2 LEPs ↑ attachment avoidance ∞↑ local peak amplitude of N2 LEP
**Social feedback**	***Social conformity***	Yoshida et al. (40)	17 HC (8M|9F)	*Cue observation from others (stimulus-related*) Mean score—below or above the participant / Variance (uncertainty) – small/large	Observed mean across all conditions	↓↑ pain ratings followed observed mean	↓↑ followed the observed mean: ACC, bil AI, bil DLPFC	↑ r bil AI, ACC, bil DLPFC ∞↑ pain ratings ↑uncertainty-induced hyperalgesia ∞↑PAG ↑uncertainty sensitivity ∞↑uncertainty-induced analgesia
					High variance vs. Low variance	↑ regardless of the observed mean	↑ PAG	
		Koban et al. (41)	36 HC (16M|20F)	*Cue observation from others (stimulus-related*) Conditioned—learned cues of low or high intensity / Social—others' ratings higher or lower than the participant	Social/Conditioned cues high vs. low	↑pain ratings	↔ NPS ↔ SIIPS	N.A.
					Social vs. Conditioned cues (high vs. low)	↑pain ratings ↑SCR	N.A.	SCR was only modulated by Social information (and not by Conditioned cues)
					Social cues high vs. low	↑pain ratings	↑ pain-related areas (ACC, bil AI, thalamus), bil DLPFC, l Amyg, IPS	Mediators of social information on pain (social high>low): ↑bil VLPFC, DMPFC, bil DLPFC, l IPS, visual cortex ↑ frontoparietal & dorsal attention networks, associated with cognitive control
	***Empathetic feedback***	Fauchon et al. (42)	30 HC (16M|14F)	*Listening to auditory comments abou*t *participants' pain attitude* Empathetic/Neutral/Unempathetic comments	Empathetic vs. Neutral	↓pain ratings	↑ r AI, r PPC, r DLPFC ↓ l MFG ↑FC: VMPFC-AI, VMPFC-PI, PI-AI ↓FC: VMPFC-PCC	N.A.
					Empathetic vs. Unempathetic	↓pain ratings	↑ PCC, Precuneus ↑FC: VMPFC-AI ↓FC: VMPFC-PCC	
					Unempathetic vs. Neutral	↔pain ratings	↑r AI ↓ VMPFC, PCC, Precuneus ↑FC: PI-AI	
**Group membership**	***Social exclusion***	Bungert et al. (43)	20 BPD patients (20F) 20 HC (20F)	*Cyberball task* with Exclusion/Inclusion / Control (instructed motor response)	HC/ BPD: Exclusion vs. Inclusion/CTRL	↑pain ratings	↑ l AI, r thalamus, r Amyg	HC_Exclusion: ↑ r Amyg ∞↑ pain ratings
					BPD (vs. HC) in Exclusion	N.A.	↑ r PI	N.A.
		Bach et al. (44)	17 OMT patients (16M|1F) 21 HC (19M|2F)	*Cyberball task with* Exclusion/Inclusion/Control (instructed motor response)	HC/OMT Exclusion vs. Inclusion/Control	↑pain ratings ↑exclusion rating	N.A.	No effect of partnership status on exclusion or inclusion conditions. HC/OMT_Exclusion: ↑ bil Amyg, AI ∞↑ pain ratings
					HC Exclusion vs. Control	↑pain ratings ↑exclusion rating	↑ pain-related brain areas (ACC, MCC, bil AI, bil thalamus), bil caudate, bil MFG, bil VMPFC, PCC, Precuneus	
					HC Inclusion vs. Control	↓pain ratings ↑inclusion rating	↑ MCC, PCC, Precuneus	
					HC Exclusion vs. Inclusion	↑pain ratings ↑exclusion rating	↑ ACC, l OFC, bil caudate, bil MFG, bil MTG	
					OMT vs. HC Inclusion	↔pain ratings ↑ exclusion ↓ inclusion	N.A.	
		Landa et al. (45)	20 HC (10M|10F)	*Cyberball task* with Acceptance/Rejection/Re-Acceptance	Rejection vs. Acceptance	↑pain ratings ↑rejection-related feelings	↑ pain-related brain areas (bil AI, r thalamus), pons ↓ MCC, bil MTG, l IPL,	Rejection: ↑ exclusion feeling ∞↑ r AI Acceptance: ↑ exclusion feeling ∞↑ pain ratings, ↑ l AI Re-Acceptance/ Acceptance: ↑ perceived rejection ∞↑ pain ratings
					Re-Acceptance vs. Acceptance	↔pain ratings ↑rejection-related feelings	↑ bil AI, r thalamus, pons ↓ pain-related brain areas (ACC, bil PI, r SI) r Amyg, bil MFG, Precuneus	N.A.
	***Stereotypes***	Schwarz et al. (46)	34 HC (34M)	*Stereotype priming* Men are less sensitive to pain (MLPS) / Women are less sensitive to pain (WLPS) / No priming (CTRL)	Across all conditions	Pain ratings: MLPS < CTRL < FLPS		N.A.
					MLPS/FLPS vs. CTRL	↔pain ratings		N.A.
					FLPS vs. MLPS	↑pain ratings	↑ ACC, r PI, bil thalamus, bil NAc,	N.A.
					MLPS vs. FLPS	↓pain ratings	N.A.	MLPS: ↓ l NAc ∞↓ pain ratings
	***In/Out group***	Hein et al. (47)	36 HC (36M)	*Receiving pain relie*f *(treatment)* from Ingroup member (Swiss)/Outgroup member(Balkan ethnicity)	Outgroup member: Post vs. Pre treatment	↓pain ratings	↓bil AI, l SI	↑ learning signal in r AI ∞↓ impression ratings, ↓ pain ratings r AI mediates social impression effect on pain ratings.
					Ingroup member: Post vs. Pre treatment	↔pain ratings	↔ bil AI	N.A.
						↓pain ratings ↓impression rating (of treatment provider)		N.A.
					Outgroup vs. Ingroup		

*Please note, the neuroimaging results reported in the table are the ones during the application period of painful stimuli—unless otherwise noted.

Ref., Reference; ∞, Correlate; ↑, Increase; ↓, Decrease; bil, bilateral; r, right; l, left; d, dorsal; M, Male; F, Female, N.A, Not applicable, result or analysis was not provided; FC, Functional connectivity, HC, Healthy controls.

Anterior cingulate cortex (ACC), Anterior insula (AI), Amygdala (Amyg), Borderline personality disorder (BPD), Control (CTRL), Dorsolateral prefrontal cortex (DLPFC), Default mode network (DMN), Dorsomedial prefrontal cortex (DMPFC), Insular cortex (INS), Inferior parietal lobe (IPL), Intermittent theta burst stimulation (iTBS), Laser evoked potentials (LEPs), Lateral prefrontal cortex (LPFC), Mid cingulate cortex (MCC), Middle frontal gyrus (MFG), Mid insula (MI), Medial prefrontal cortex (MPFC), Middle temporal gyrus (MTG), Nucleus accumbens (NAc), Neurologic Pain Signature (NPS), Orbitofrontal cortex (OFC), Opioid maintenance treatment (OMT), Periaqueductal gray (PAG), Posterior cingulate cortex (PCC), Posterior insula (PI), Posterior parietal cortex (PPC), Skin conductance response (SCR), Superior frontal gyrus (SFG), Primary somatosensory cortex (SI), Stimulus intensity independent pain signature (SIIPS), Supramarginal gyrus (SMG), Superior temporal gyrus (STG), Temporoparietal junction (TPJ), Ventrolateral prefrontal cortex (VLPFC), Ventromedial prefrontal cortex (VMPFC).

Table 1 summarizes the 19 studies that were retained after the selection process. For readability, each study's core findings and the main conclusion for each thematic group were summarized.

In consideration of a potential limitation of the search results, please note that, in the search strategy (see “Search strategy” section, *Methods*), the term “fNIRS” was not included as a keyword. It is possible that studies that were tagged in the databases with the specific keyword “fNIRS” without being also tagged with any “neuroimaging” or neuroimaging-related keyword, topical word, or MeSH term might have been missed. However, using the general keywords, many identified studies were conducted with fMRI, few with EEG, fewer with EEG-TMS, and none with PET or another neuroimaging method (see Table 1). It seems, therefore, unlikely that a sizeable number of fNIRS studies was missed, although this possibility cannot be excluded.

### Social signals' themes and their influence on self-pain

#### Helping others

Studies in this thematic group focus on situations in which an individual can help others in a certain way, before or during, the experience of a painful event. The motivation behind these studies is to explore whether the subjective experience of pain can be altered when one decides to give support to another person. Two fMRI studies showed that helping others reduces stimulus intensity rating and attenuates activation in response to painful stimuli in pain-related brain areas—either by donating to a stranger (29) or by taking the suffering from a close one (30).

In the first study, Wang et al. (29) found that when participants faced altruistic decisions and chose to donate part of their initial allowance to an orphan, they perceived subsequent electric shocks to be less painful (vs. a control condition with a matched visual decision task). It should be noted that 94% of the participants chose to donate in the donation trials; therefore, comparing the conditions could be regarded as exerting altruistic behavior vs. not (Control condition). At the neural level, the altruistic (vs. Control) condition led to reductions in response to painful stimuli in pain-related brain areas (dACC, bilateral insula [posterior insula (PI), middle insula (MI), anterior insula (AI)], right thalamus, primary somatosensory cortex [SI]), and in the right caudate, the left middle frontal gyrus (MFG), and the right inferior parietal lobe (IPL). The more the participants considered their donations helpful (measured by perceived helpfulness rating post-experiment), the more the attenuation of neural activation in the dACC and the bilateral insula was observed. Attenuating neural activation in the dorsal anterior cingulate (dACC) and the bilateral insula also correlated with increased neural activation in the ventromedial prefrontal cortex (VMPFC) at the donation phase.

Similarly, López-Solà et al. (30) reported a reduction in pain ratings (unpleasantness, but not intensity ratings) when participants chose to receive painful stimulations that were intended for their romantic partner (Accept-Partner-Pain vs. Baseline). In addition, trials in the Accept-Partner-Pain condition resulted in increased engagement in positive thoughts (vs. Baseline). Neural activation in the Accept-Partner-Pain (vs. Baseline) condition decreased in the left AI and the right orbitofrontal cortex (OFC) but increased in the right thalamus and the VMPFC. Although the Accept-Partner-Pain condition did not modulate the Neurological Pain Signature (NPS) (48), engagement in positive thoughts was positively correlated with NPS reduction. An increase in partner pain acceptance (i.e., the percentage of trials in which participants chose to accept the partner's pain) correlated with decreases in pain ratings, increased neural activation in the VMPFC, and increased engagement in positive thoughts. A whole-brain mediation analysis was used to interrogate brain areas mediating the effect of accepting the partner's pain on pain ratings. The analysis showed that neural activation reduction in the midcingulate cortex (MCC), bilateral AI, SI, the right lateral prefrontal cortex (LPFC), and OFC predicts the reduction in pain ratings and the NPS. Finally, reductions in the neural activation of pain-related areas (bilateral AI, MCC, SI) were mediated by an increase in neural activation in the VMPFC and a decrease in the right OFC.

### Egocentric interpersonal perceptions

This theme focuses on experiments exploring how specific interpersonal actions such as observation (31) and evaluation of others' feeling states (32) can directly influence individuals' pain experiences. The first study (48) measured pain using laser-evoked potentials (LEPs).

The functional roles of pain evoked potentials have been widely explored in neurophysiological studies delineating four main components (N1, P1, N2, and P2), of which the amplitudes and latencies are modulated by different experimental manipulations [see Chen et al. (49) for a detailed overview]. Specifically, the early components N1-P1 (~100 ms latency) have been shown to reflect activations from the operculo-insular cortex and SI and therefore are interpreted to be associated with the sensory processing of pain (50). Later components such as N2-P2 and P3 seem to originate from brain areas, such as the AI and ACC, and are thus thought to reflect affective pain processing (51). However, it has been posited that none of the LEPs are specific to pain but reflect a more general, salience-related processing of the noxious stimulus (52).

Using laser-evoked potentials (LEPs), Valeriani et al. (31) investigated how being in pain can be affected by observing others in pain. In their study, while receiving a painful stimulus on the hand, participants watched several videos differing in contexts regarding the nociceptive potential of a stimulus (Needle/Q-tip/None) applied to a specific model target (a person's hand, a person's foot, a tomato). Participants were asked to rate their pain from the stimulus, the movie (self-referred pain), and the model (other-referred pain). The study found that none of the observation contexts modulated participants' stimulus pain ratings. As for the referred pain ratings, however, observing a needle penetrating another's hand or foot (vs. all other conditions) resulted in increased self- and other-referred pain ratings. Also, participants rated the stimulus pain higher than the self-referred pain but lower than the other-referred pain. At the neural level, modulations were observed only with the Needle in Hand condition (vs. all other conditions). Specifically, the amplitudes of the N1 and P1 LEP components [associated with sensory processing of pain (50)] were decreased, and there was no effect on the N2/P2 LEP components [associated with affective processing of pain (51)]. Furthermore, an increase in the difference between self and other referred pain ratings correlated with a decrease in the N1/P1 LEP components. That is, the more participants rated the pain induced by the movie higher in themselves than in the model, the greater the reduction in the amplitude of the N1/P1 LEPs. Lastly, although there was no direct effect of the observation context on stimulus pain ratings, an indirect effect was found: a *post-hoc* analysis revealed a positive correlation between self-referred pain ratings and the stimulus pain ratings.

In 2020, Ellingsen et al. (32) published an fMRI-hyperscanning experiment investigating pain-related social effects of a clinician-patient interaction. In the study, chronic pain patients (diagnosed with fibromyalgia) were connected to an electroacupuncture (EA) device while in a scanner. Pairs of patients and clinicians could see the face of each other during the experiment *via* MRI-compatible cameras that were attached to a table-mounted mirror on each MRI scanner and manually adjusted to capture the entire face.

During the task, participants received painful stimuli (pressure evoked pain) after an anticipatory period with cues predictive of the clinician's decision whether to execute (or not) the EA treatment during the pain delivery to the participant. Two manipulations were tested for their influence on participants' pain experience: execution of the EA treatment (vs. no EA treatment) and patient-clinician interaction (vs. no interaction). The interaction condition was in the form of a brief clinical intake interview with the clinician (done on a separate day before the scanning), which ended with the requirement for both to rate their perceived relationship with each other during the intake (therapeutic alliance ratings). In addition, the two EA conditions were compared against a Sham condition. During the task, pain ratings (by participants) and vicarious pain ratings (clinicians' estimated rating of participants' pain) were collected, as well as the perceived relationship of a patient/clinician with each other during the scanning session (relationship quality ratings). Comparison between the EA (vs. sham) treatment on pain ratings revealed no significant effect, and therefore, both trial types were grouped together as treatment conditions in subsequent analyses. The treatment (vs. no treatment) condition reduced participants' pain ratings and clinicians' vicarious pain ratings and increased patients' and clinicians' positive feeling ratings. Moreover, the decrease in participants' pain ratings correlated with a decrease in clinicians' vicarious pain ratings but an increase in clinicians' accuracy in treatment-efficacy estimation (i.e., the degree of correlation between patient's vicarious pain rating with participants' pain rating before/after treatment) and in relationship quality scores. Further, stronger treatment-related analgesia was reported by participants with higher therapeutic alliance ratings. Interestingly, during the anticipation period of participants, increased facial mirroring between participants and clinicians positively correlated with an increase in treatment-related analgesia (decrease in pain ratings) and therapeutic alliance scores. The more the participants and their clinicians mimicked each other's facial expressions during the anticipatory phase, the better the participants perceived their relationship with the clinicians and the stronger the feeling of analgesia they experienced. Participants' neuroimaging results showed increased neural activation in the prefrontal regions (ventrolateral prefrontal cortex [VLPFC], medial prefrontal cortex (MPFC), and bilateral DLPFC), the left superior temporal sulcus (STS), and the bilateral temporoparietal junction (TPJ) in the treatment (vs. no treatment) condition. Specifically, the reduction in pain ratings positively correlated with increased neural activation in the right VLPFC, precuneus, the IPL, and the supramarginal gyrus [SMG]. The study found no effect of the Patient-Clinician interaction (a brief interview) on the subjective pain ratings.

### Social support

Within the research literature investigating social effects on pain, social support is the most explored theme. In this theme, the feeling of support in individuals undergoing painful experiences is often induced experimentally by asking the participants to view a photo of their romantic partner, feel their touch, or simply inform them about their presence. Hence, social support can be achieved by relatively passive (viewing, general presence) or active (affective touch) means. Seven studies were identified in the search, sub-grouped by the support induction method: social viewing, affective touch, and social presence.

#### Social viewing

Two fMRI studies (33, 34) and one brain stimulation study with EEG (35) explored how viewing the photo of a romantic partner while receiving a noxious stimulus can alter participants' pain perception (relative to viewing a photo of an acquaintance or engaging in a distracting task).

Younger et al. (33) found that viewing a partner's photo or engaging in a distraction task while being in pain reduced pain ratings (relative to viewing a photo of an acquaintance). Comparing the analgesic effect of the two conditions (partner viewing vs. distraction task) on behavioral ratings revealed no significant difference. Hence, both tasks reduced pain ratings with a similar magnitude. In contrast, examination of the neuroimaging data revealed differences in the recruitment and modulation of specific brain areas. Viewing the partner's photo during pain (vs. all other conditions) reduced neural activation in pain-related sensory areas (bilateral PI, thalamus, SI) and the right DLPFC. Moreover, partner-related analgesia (reduced pain ratings) also correlated with decreased neural activation in pain-related affective processing areas [left AI and anterior-dorsal part of the ACC (adACC)]. Increased neural activation during partner viewing (vs. all other conditions) was observed in the subgenual ACC (sgACC), MCC, bilateral OFC, left amygdala, and precuneus. Furthermore, partner-related analgesia correlated with increased neural activation in a cortical network that is associated with reward processing (e.g., bilateral nucleus accumbens [NAc], bilateral caudate, bilateral OFC, left amygdala) (53–56), as well as with the right DLPFC and the right thalamus. Similar analysis showed that distraction-related analgesia was correlated with increased activation in the pregenual ACC (pgACC), the bilateral OFC, the left DLPFC, and the left MFG. The only significant functional overlap was seen in the right OFC, which positively correlated with partner and distraction related analgesia.

Using a similar paradigm, Eisenberger et al. (34) investigated how viewing a partner's photo can influence participants' experienced pain. Differently from Younger et al. (33), the control viewing conditions included either a photo of a stranger or an object, and the neuroimaging investigation focused on two structural regions of interest (ROIs) associated with physical pain—the dACC and bilateral AI, and functional ROIs discovered in the contrast partner's (vs. stranger/object) photo viewing—the VMPFC and the premotor cortex. Furthermore, the study also tested the modulation effects of two trait measures: perceived partner support and relationship duration. The behavioral results showed reductions in pain ratings in the partner (vs. stranger/object) condition. No difference in pain ratings was found between the stranger and object conditions, so they were collapsed into one Control condition. Viewing the partner's photo (vs. stranger/object conditions) was also accompanied by reduced neural activation in the two pain-related ROIs (dACC and bilateral AI) and increased activation in the VMPFC and premotor cortex. This increase in VMPFC activation was correlated with decreased neural activation in the dACC, as well as with higher ratings of perceived support and longer relationship duration. Finally, reductions in pain ratings correlated with increased neural activation in the VMPFC and decreased activation in the dACC.

The third study on this theme applied the same photo-viewing task described in the previous studies. However, it offered a causal (rather than correlational) examination of neural activation and network connectivity by applying a facilitatory intermittent Theta Burst Stimulation (iTBS) on the left dorsomedial prefrontal cortex (DMPFC). Specifically, gamma-band activity has been suggested to encode the subjective pain experience (57, 58). The effects of iTBS on behavioral ratings, neural activity, and network connectivity using EEG were examined. In this study, Che et al. (35) found that partner's (vs. stranger) photo viewing during pain delivery reduced pain ratings (before applying iTBS) and correlated with increased perceived support ratings. Applying the iTBS further increased the reduction in pain ratings in the partner (vs. stranger) condition. Within the partner condition, examination of the iTBS effect (partner condition: pre vs. post iTBS) resulted in no change in pain ratings but increased the fronto-central gamma activity, increased the connectivity between frontal and occipital regions, and decreased perceived support ratings. In comparison, iTBS in the stranger condition increased pain ratings, central-parietal gamma activity, and connectivity between central and frontoparietal regions but did not change the perceived support ratings. Finally, a source estimation analysis using TMS-EEG showed that the increased gamma activity was found to be correlated with increased pain-related N100 amplitude.

#### Social touch

Three studies (36–38) investigating the effects of social support on pain modulation used a more active approach to induce support in participants undergoing pain—a supportive, tactile touch (termed “social touch”). Two of these studies (37, 38) examined changes in pain perception when participants held hands (static touch, without movement) with their romantic partner, with a stranger, or held an object. The third study examined participants' pain when they held hands with their romantic partner in either a slow-affective or a fast-neutral manner (dynamic touch, with movement) (36).

Consistently with the effects reported by the studies using social viewing described above, social touch by a romantic partner (vs. control conditions) was found to increase emotional comfort (38) and decrease pain ratings (36–38), the NPS (38), and activity in brain areas (37, 38) and evoked potentials (36) associated with pain processing.

A particular insight into the mechanisms underlying social touch analgesia comes from the study of von Mohr et al. (36). This study examined what type of touch is effective in reducing pain. By changing the pace of the partner's touch, the results show that, even when coming from the partner, the supportive touch has to be slow (i.e., “affective”) rather than fast (i.e., “neutral”) in order to lead to reductions in pain ratings and related neural processing (decreased local peak amplitudes of N1, N2, and P2 LEPs). Moreover, the study also found a significant interaction between attachment anxiety and pain ratings, indicating that higher attachment anxiety scores lower the pain rating difference between slow and fast touch.

Interestingly, Kreuder et al. (37) found that social support received by holding the hand of either a romantic partner or a stranger reduced pain unpleasantness ratings (vs. no support). However, when comparing the two support conditions, being touched by a partner leads to stronger analgesia than being touched by a stranger. The neuroimaging results showed that both partner and stranger support (vs. no support) reduced the pain-related activation in the left AI. Contrasting these two conditions demonstrated increased neural activation in the right MFG in the partner (relative to the stranger) support condition. As Kreuder et al. tested both men and women, they examined gender-specific neural activation and found differences across the conditions that occurred only for female subjects: relative to no support, increases in neural activation were found in the left thalamus and the left caudate with partner support and in the VMPFC and the left amygdala with the stranger support. Comparing the two support conditions showed increased activation in the VMPFC with the partner (vs. stranger) support. These results suggest a gender-specific difference in the neural modulation of pain by social support.

Lastly, López-Solà et al. (38) found that holding the hand of a partner (vs. object holding) reduced pain ratings and decreased neural activation in pain-related brain areas (ACC, left AI, left thalamus), the prefrontal areas (bilateral MPFC, bilateral DLPFC, bilateral OFC), the left amygdala, the periaqueductal gray (PAG), and the SI. Moreover, partner support also reduced NPS activation (but was not correlated with the reduction in pain rating). It increased connectivity between the NPS and the primary sensory cortex (SI), the default mode network (DMN) regions (MPFC, posterior cingulate cortex [PCC], precuneus), the NAc, and the middle temporal gyrus (MTG). A whole-brain multi-level mediation analysis revealed that the most potent mediators of the observed touch-induced analgesia were activation reductions in prefrontal brain areas (DLPFC, VLPFC, DMPFC, VMPFC), OFC, amygdala, ACC, and PAG. Finally, the results showed that increased emotional comfort ratings correlated with reductions in pain ratings (during the partner condition) and increases in perceived relationship quality scores.

#### Social presence

The last study on this theme showed how the mere presence of a person could affect the individual experience in a counterintuitive way. Krahé et al. (39) showed that informing participants experiencing pain about the presence of their loved one (in the same room) did not affect their ratings (relative to when the partner was absent) but increased the peak amplitude of pain-related LEP components (increased P2 local peak amplitude of the P2-N2 complex). The study also compared conditions in which participants were told about the partner's presence and their focus—the partner either focused on the participant being in pain or on the ratings of another participant. No difference was found between these two focus conditions. In the partner presence (vs. absence) condition, higher attachment avoidance scores correlated with increases in pain ratings and local peak amplitudes of N2 and P2 LEPs. Regardless of the partner's presence, attachment avoidance scores positively correlated with the increase in local peak amplitude of N2 LEP. Finally, higher attachment anxiety scores correlated with decreases in the latency of N1 and N2 LEPs.

#### Social feedback

As the previous section shows, familiarity and closeness in social interactions can significantly influence the individual pain experience when receiving support. In other social contexts, unfamiliar strangers can also shape individuals' perceptions of pain. This section reports on three papers (40–42) that explored how different forms of feedback from strangers can modulate the pain experience (i.e., social feedback effects). In the first two studies (40, 41), social conformity manipulation was employed to test how others' evaluations of a painful event might alter the individual's self-experience of a similar event.

In a study by Yoshida et al. (40), participants were shown stimulus pain ratings of a group of strangers who experienced the same stimulus beforehand. The group ratings were shown as a distribution line graph, characterized by a specific mean (below or above the participant rating) and variance (small/large) values. Consistent with conformity studies, the behavioral results showed that the ratings of others influenced participants: participants' pain ratings followed the experimental group means (in both directions). Accordingly, the observed mean modulated neural activation in the bilateral AI, the ACC, and the DLPFC, which was correlated with pain intensity. Interestingly, it was found that high (vs. low) variance increased participants' pain ratings—regardless of the observed mean. This uncertainty-induced hyperalgesic effect correlated with neural activation in the PAG.

Koban et al. further demonstrated the strong influence of social conformity on an individual's pain perception (41). In their study, participants were presented with two cues predictive of the intensity of upcoming painful stimulations. The first cue presented the pain ratings of other people (social cue), while the second cue displayed a photo that was conditioned, before the task, to a specific pain intensity (conditional cue). The authors found that both cues modulated expectancy and pain ratings in line with the predicted information (high/low intensity). However, stronger cue effects (i.e., greater increase/decrease) on the subjective ratings were observed with the social cues (vs. conditional cues). Moreover, the study found that social information (but not conditioned learning) increased skin conductance responses during painful stimulation. The neuroimaging data revealed that social cues of high (vs. low) pain increased neural activation in pain-related brain areas (ACC, AI, thalamus), as well as areas involved in somatosensory integration (MI, parietal operculum), emotion processing (amygdala), cognitive control and top-down attention modulation (DLPFC, IPL, and IPS). In contrast, different neural structures were associated with the modulation of conditional cues on pain (e.g., hippocampus, caudate, cerebellum). A mediation analysis revealed that the brain regions contributing most to mediating social information on pain ratings were the DLPFC, the DMPFC, the VLPFC, the IPS, and the visual cortex. Interestingly, neither the social information nor the conditioned learning directly affected the two neural signatures associated with pain that was tested in the study—the NPS and the stimulus intensity independent of pain signature (SIIPS). Instead, both effects were mediated by expectancy ratings (acquired before the stimulus).

The studies above illustrate how social feedback (presented as ratings of similar experiences by unfamiliar others) significantly impacts the individual's pain experience. However, the nature of information, i.e., the group's perceived pain intensity, is often not visible or easily disclosed to individuals in everyday life. Another type of social feedback that is more common in a natural setting concerns signals from another person (a stranger, a clinician, etc.), such as direct comments or expressions about the state of individual suffering. The final study on this theme by Fauchon et al. (42) varied the content of auditory comments by a stranger regarding the participant in pain. The behavioral results show that only participants who heard empathetic comments about their suffering rated the pain stimuli less intense (vs. neutral or unempathetic comments). Between the neutral and unempathetic comments, no significant difference was found. During pain, empathetic (vs. neutral) comments increased neural activation in the right AI, the right DLPFC, and the right posterior parietal cortex (right posterior parietal cortex [PPC]), and decreased activation of the left MFG. In the unempathetic (vs. neutral) comments condition, neural activations in the rAI and the PPC were increased and decreased in the VMPFC and thePCC/precuneus. Finally, connectivity analysis revealed that, in the empathetic (vs. neutral/unempathetic) comments condition, functional connectivity increased between VMPFC-AI and VMPFC-PI and decreased between VMPFC-PCC.

### Group membership

In the previous theme about social support, it became clear that the quality of a romantic relationship can influence pain modulation by support. A related yet distinct topic of investigation focuses on investigating pain modulatory effects stemming from a relationship with a group. This relationship can be very brief, and the group members can be utterly unfamiliar with the individual. For this theme, five studies (43–47) were identified in the search. The first three studies (43–45) investigated group membership effects using a computer game (Cyberball), triggering the individual's experience of inclusion or exclusion from a group. The two other studies explored how inherent in- and out-group perceptions about others (47) or oneself (46) change pain-related perceptions and processing.

#### Social exclusion

Consistently with behavioral studies on social exclusion and pain, three recent neuroimaging studies (43–45) found that, after healthy participants were excluded (vs. included/control condition) in the Cyberball game, they perceived fewer interactions, rated subsequent pain stimulations as more intense, and felt more excluded, rejected, ignored, and invisible (45). A hyperalgesic effect of social exclusion has also been observed in patients with borderline personality disorder (BPD) (43) and in patients on opioid maintenance treatment (OMT)(44). The interpretation of the latter two studies is beyond this review's scope because it focuses on healthy participants and chronic pain patients. The reported results nevertheless show the consistency of the finding across different populations.

Reviewing the neural activation evoked during pain after social rejection (vs. inclusion) across the three studies on healthy participants revealed consistent activation increases in the insula and the thalamus in response to painful stimulation: the AI [left AI (43), the bilateral AI (44, 45)], and the right thalamus (43–45). Within the cingulate cortex, the results were less consistent, and included neural activation that increased in ACC and MCC (44), decreased in MCC (45), or did not change significantly (43). Moreover, Bungert et al. (43) also observed increased neural activation in the right amygdala in the social exclusion condition. As for parametric modulations, Bach et al. (44) found that subjective pain ratings positively correlated with neural activation in a cluster that included the bilateral AI, the hippocampus, and the amygdala during social exclusion. Only within the exclusion condition, a positive relationship between neural activation and pain ratings in the amygdala was also observed by Bungert et al. (43). Finally, Landa et al. (45) found that, among a set of Interpersonal emotions (exclusion, rejection, ignoration, feel invisible, feel liked) and non-specific emotions and comfort (feel good, feel comfortable, feel powerful), only exclusion ratings correlated with neural activation in the right AI.

The study by Landa et al. (45) introduced a new Cyberball condition in their experiment—“re-acceptance,” which was always presented after participants had undergone the rejection condition. During the re-acceptance condition, the other players renewed the individual's membership in the group by including them in the game again. It was observed that, even after the rejection condition had ended, feelings of exclusion persisted: participants felt more excluded, rejected, and ignored (comparing re-acceptance vs. acceptance). Moreover, the more the participants felt rejected during reacceptance, the more intense they felt the painful stimulus (higher pain ratings). However, in contrast to the rejection condition (vs. acceptance), the reacceptance (vs. acceptance) condition showed decreased neural activation in pain-related (bilateral PI, ACC) and affective brain areas (amygdala, MTG) but increased activation in the pons.

#### Stereotypes

Whereas social exclusion tasks are manipulations in which other individuals actively dictate the status of an individual's relationship with a group (by accepting/rejecting an individual to/from the group), a study conducted by Schwarz et al. (46) primed male participants with a gender-specific stereotype about pain to allow them to join a “conceptual group” (by believing the stereotype). Specifically, male participants who were primed with the information before the experiment that “males are less sensitive to pain” (MLPS group) showed decreased pain intensity and increased heat pain thresholds (vs. control group with no priming). The exact opposite effects were found when another group of male participants was primed with the information that “Females are less sensitive to pain” (FLPS group) relative to a control group. The stereotype-based priming modulated the pain processing, suggesting that the behavioral effects are unlikely to be caused solely by response bias: FLPS (vs. MLPS) priming led to increased neural activation mainly in pain-related brain areas (ACC/MCC, right PI, thalamus) and bilateral NAc. A correlation between neural activations and pain ratings was found only in the MLPS priming group, where a decrease of neural activation in the left NAc was observed (compared to testing the MLPS group without priming) associated with lower pain ratings.

Finally, the authors tested the effect of individuals' perceived masculinity (acquired as trait ratings) on pain ratings but found no significant correlation. The results suggest that stereotypes about pain can alter both the subjective experience and the neural processing of pain, adhering to the stereotype contextual direction.

#### In-group/out-group effects

In a recent study by Hein et al. (47), the authors investigated whether in/out-group exert their effects directly on pain perception or indirectly *via* influencing pain-relief learning (i.e., learning from cues/individuals associated with pain-relief). Therefore, following a classical conditioning paradigm in which a visual cue was associated with an upcoming painful stimulus, participants had to learn a new association during “treatment sessions”. In those sessions, the cue was primarily associated (75% of the time) with a pain-relief treatment, which was achieved by omitting the painful stimulus from either an in-group or out-group member referred to as the “treatment provider” (a confederate). Participants were only told that the treatment provider would make decisions that could affect their pain stimulation. The group membership manipulation was executed by letting the treatment providers introduce themselves to participants with their full names before the treatment session. The names indicated whether they were of the same (Swiss) or different nationalities (Balkan descent) as the participant. The out-group nationality was picked to be a minority in the study's country and against which the local population held a negative prejudice). After the short introduction, participants rated their impressions of the in- and out-group members. The social manipulation was validated by showing that the out-group members were rated significantly more negatively (vs. in-group members) on perceived group membership, similarity, and likability.

The behavioral data showed that learning (captured by changes in ratings of anticipated emotions during the treatment period) occurred in the in- and out-group treatment conditions without any difference in learning rate. Pain-relief learning was reflected by neural activation in the AI (mostly the right AI). Somewhat counterintuitively, pre-to-post treatment analysis showed that the out-group, but not in-group, treatment condition led to reductions in pain intensity ratings and pain-related neural activation (left AI, SI). A mediation analysis revealed that the analgesic mechanism was learning-based and mediated by the rAI. That is, increased neural activation in the rAI of the out-group condition correlated with larger reductions in pain ratings. Finally, it was observed that the more negative impression participants gave about the out-group member, the greater the analgesic effect they exhibited on pain ratings and pain-related processing.

## Discussion

### Main summary

**Figure 2:**
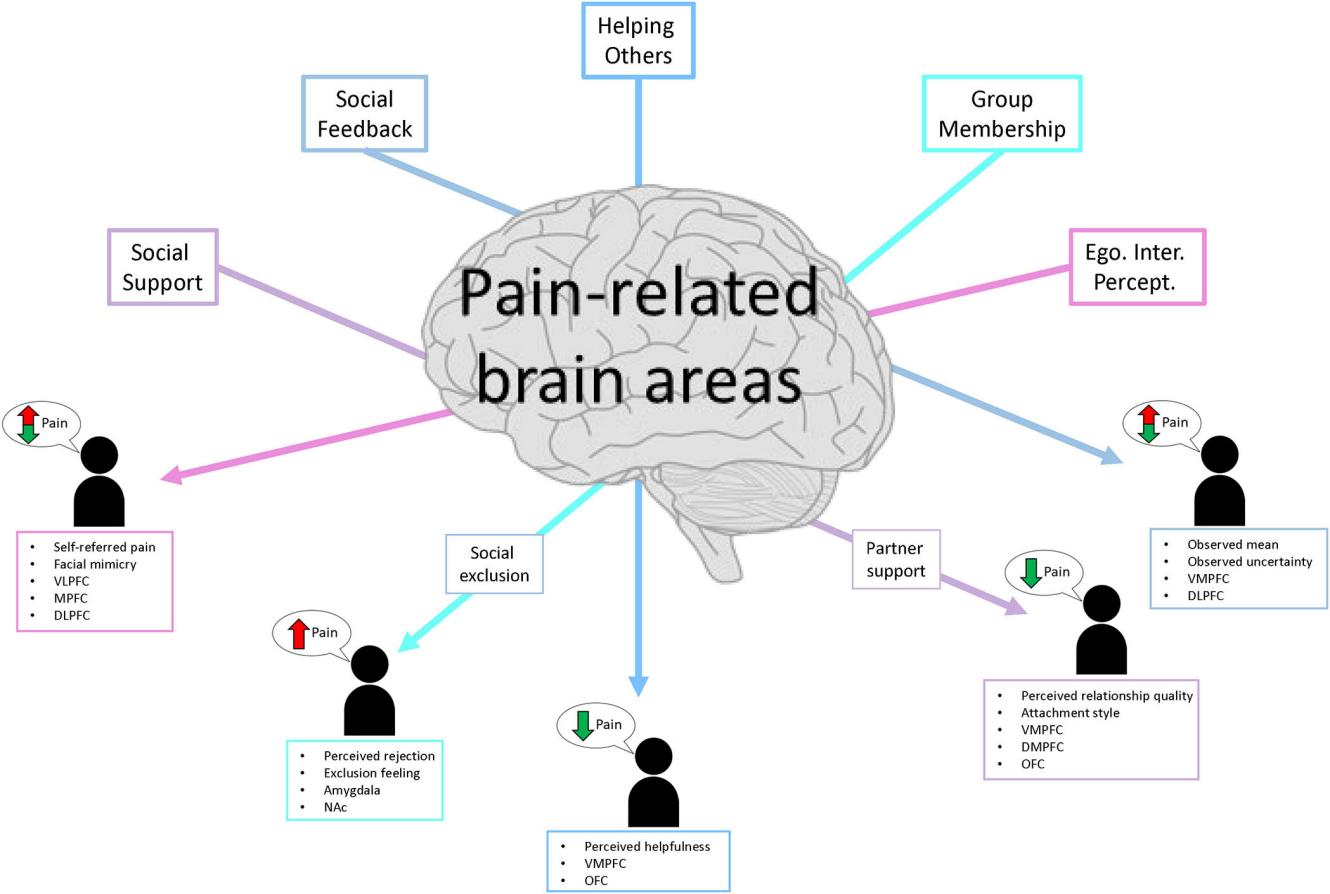
Sketch summary of the five social themes, the direction of their influence on pain ratings, and the main modulatory/mediating brain regions.

The studies included in this review examined how different social manipulations changed the experience of pain and pain-related effects using different readout measures (e.g., pain ratings, emotion ratings, decision-making, physiological signals, and changes in neural activity) in a controlled lab environment. The findings will be synthesized in the following sections, focusing on overlapping and distinctive processes and the neural mechanisms that contribute to pain perception and processing modulation. Finally, a conclusive summary of the reviewed topic is also provided (see Figure 2 for a summary sketch).

### Social signals alter subjective pain ratings and pain-related neural processing

Across the studies reviewed here, social manipulation altered the individuals' pain ratings in 16 out of 19 studies. In three studies, social manipulation did not modulate pain intensity ratings. These include an fMRI study within the “Helping Others” theme (30) and two EEG studies within the “Egocentric Interpersonal Perceptions” (31) and “Social Support” themes (39). Two of these studies (30, 31) also assessed pain unpleasantness ratings, but only one on social modulation was observed (30). Across the 19 studies, 14 studies only measured pain intensity, two studies measured only pain unpleasantness, and three studies measured both.

Examining the neuroimaging results during periods when painful stimuli were administered confirms that social manipulations modulated neural activation in pain-related brain areas in 14 of the 15 fMRI studies. The most consistent modulations across the themes were located in areas primarily associated with the sensory (thalamus, SI) and affective processing of nociceptive signals (AI, ACC). Similar modulations were also observed in areas associated with pain modulation (VMPFC, DLPFC) and affect processing (amygdala). Clustering the results of studies that performed correlation analysis with subjective reports revealed that neural activation in the AI was most frequently (6 studies) correlated with changes in pain ratings due to social manipulations [all positive correlations, higher (or lower) AI activation correlated with higher (or lower) pain ratings, respectfully)], followed by the ACC, the VLPFC, the DLPFC, and the amygdala (4 studies each). Only one fMRI study (32) did not show modulation of activation in pain-related brain areas, which might be because the experiment did not have a control condition without pain but a treatment and a no-treatment condition, both with pain.

Across the four evoked related potential (ERP) EEG studies reviewed here (mainly using laser-evoked potentials [LEPs]), social manipulations on the N100 pain-related LEP component were observed only in three studies either directly (31, 36) or indirectly (35). Other LEPs were found to be modulated either one time (P1, N2) (31, 36) or two times (P2) (36, 39) across the four studies. Concerning a relationship between pain-stimulus ratings and specific LEP components, either no correlation was found (31, 35) or such correlation was not tested (36, 39). The absence of a relationship between pain-stimulus ratings and the LEP component is consistent with the pain neurophysiology literature, which shows that LEPs are a good indicator of the occurrence of pain but are weak predictors of subjective pain ratings (49, 59, 60).

### Shared and distinct features of social modulations on pain perception

The findings within each theme are now examined to understand better the underlying mechanisms of social modulations on pain and the roles of modulating factors and mediating brain areas.

In the *helping others* theme, two studies examined pain perceptions and processing changes after helping a stranger (29) or a close romantic partner (30). The shared features seem independent of the help's target, helping others reduce neural activation in the AI (associated with affective pain processing during painful stimulations) and pain ratings. In terms of modulations, elevated helping-related feelings, i.e., increased feelings following the decision to help [higher perceived helplessness (29) or positive thoughts (30)] decreased both sensory and affective pain-related neural activation [reduction in NPS (30) and dACC, PI, and AI (29)]. Moreover, the more the individuals chose to help others, the higher the level of activation observed in the VMPFC—shown by a mediation analysis (30) or indirectly by a series of separate correlations (29), and the better they felt about it [higher help-related feelings (29, 30)]. In both studies, VMPFC increased neural activation was associated with decreased pain-related activation (both affective and sensory). These findings suggest that helping another person—regardless of whether they are familiar—reduces pain, which seems to be mediated mainly by the VMFPC.

Nonetheless, the two studies also had distinct features. Helping a total stranger (vs. romantic partner) (29) decreased a larger cluster of pain-related neural activation, which also included brain areas associated with the sensory processing of pain (e.g., SI, PI, thalamus). Helping a romantic partner (30) reduced mainly neural activation related to affective pain processing (reduction in AI, unchanged NPS, increase in the thalamus). This is also consistent with the observed difference in the reduction of pain intensity in the study of Wang et al. (29) but not in the study of Lee et al. (30), where only unpleasantness was decreased. Finally, in addition to the mediation by the VMPFC discussed above, helping a familiar person appears to be mediated by the OFC (as seen by mediation analysis showing that greater activation reductions in the OFC predicted reductions in pain ratings neural activations of pain-related brain areas) (30).

In the e*gocentric interpersonal perceptions* theme, one EEG study (31) and one fMRI (32) study investigated how observing or interacting with others affects the individual during pain. Despite the two different neuroimaging modalities, several shared features could be extracted. While in pain, viewing photos of human/non-human parts or a live video of a clinician before deciding to give a treatment did not affect pain ratings. However, pain ratings increased when there was more detachment from the other person. Participants rated the painful stimulus higher when they focused much more on themselves than on the observed others (31). Similarly, stimulus pain ratings increased when participants exerted less facial mimicking of the other [a form of social bonding (53)]. Finally, observing a human model or another person in pain decreased pain-related LEPs (31) and increased pain-modulating areas (32).

Examining the differences between the two studies also revealed distinct outcomes. When the location of the delivered painful stimulus (the dorsum of the right hand) matched the stimulus location of the observed person in the video, LEPs associated with sensory processing of pain (N1, N2) were reduced while stimulus pain ratings were not affected (31). In contrast, observing a person (a clinician deciding whether to give treatment or not) affected pain intensity ratings but not pain-related brain areas. Instead, it affected pain-modulating brain areas (32). These two studies illustrate how pain perception can be modulated by observing or mimicking others' actions. Moreover, the findings show that even brief interpersonal encounters can significantly impact treatment effectiveness, for which prefrontal regions (VLPFC, MPFC, and bilateral DLPFC) appear to be the critical pain-modulating players. Finally, it seems that inferring about others' feelings in relation to oneself (i.e., self-referred pain) can affect one's pain perception and processing.

Examining the *social support* theme, this review's largest cluster of studies revealed that pain ratings decreased by social support from a romantic partner in six of the seven studies. Moreover, the more concrete the partner support was, the stronger the observed modulations of pain. The strongest modulations were seen by social support given by touching or handholding (36–38), relative to those by viewing a partner photo (33–35). The weakest modulation was seen in a study in which participants were notified of the presence or absence of their partner without being able to see them (or any photo of them) (39). In this study, pain ratings were not affected directly by the social manipulation but only when attachment styles were included as covariates (39).

Based on the findings of (35), it is plausible to assume that visual processing related to social support might be essential to the modulation of partner support on pain and might explain the lack of change in pain ratings when participants do not see their partner during the painful experience. In future studies, one could examine such requirements by including belief scores, which measure the degree to which participants believed their romantic partner was present/observing them during the pain delivery (or during the whole task).

Regarding neuroimaging, partner support modulated neural activation associated with sensory (33, 35–38) and affective (33, 34, 36–39) pain processing. Similar to the behavioral results, the EEG study investigating social presence effects showed no reduction in any LEPs associated with pain (but rather an increased P2 LEPs) (39). When examining the mediating brain areas of social support on pain, prefrontal regions such as the VMPFC, the DLPFC, and the OFC again seemed to be the key players. However, this modulation depends on the relationship quality, attachment style, and individual perception of support during pain. From aggregating the findings of the social support studies, several core concepts were extracted that give insights into the mechanism of social support modulation on pain. First, for highly effective pain analgesia to occur, it is essential that the source of support would be given by “a significant other” in which the relation to the supporter has to be intimate (partner), extended (relationship length), perceived as valuable/joyful (relationship quality) and not associated with relationship fears (attachment anxiety or avoidance). Moreover, the support should be concrete (touch, viewing, rather than imagining the supporter) and with care (slow affective touch). Second, the support given by a stranger could also be beneficial (even if weaker than a supporting partner) to individuals in pain but might depend on the support modality (37). When it comes to physical touch, the intimate context of touch might provide pain alleviation irrespective of familiarity (35, 37). Alternatively, this could also manifest a distraction-based mechanism when support is given by an unfamiliar (or unexpected) stranger. This alternative is supported by the results showing a decrease in stimulus salience (like distraction) in stranger support (seen as reduced AI activation) and increased neural activation in the MFG. This region is part of the reorienting attention network (61), increasing trust toward attachment figures (seen as increased MFG neural activation) (37). Finally, evaluation of the support meaning is necessary to form a perception of the received support, which significantly influences the final analgesic effect once support is given. Taken together, partner-related analgesia might work through multiple mechanisms—encoding of the partner support as a reward/safety signal that reduces pain and pain-related stress (33), increasing the perceived support (34, 35), and shifting local and distributed network connectivity of pain-modulating brain areas (35). Finally, prefrontal regions such as the VMPFC and the DMPFC seem to be core brain areas mediating this modulation of social viewing on pain perception, where the DMPFC seems to be involved in encoding and processing the individual's perceived support.

The *social feedback* theme shows that information about a similar painful experience of others influences the direction of participants' pain ratings (40, 41). Two primary mechanistic factors can be extracted. First, it seems that an increased range of others' feedback (i.e., the variability of the social feedback) causes more uncertainty regarding deriving/learning the expected experience and, therefore, enhances the pain experience, regardless of the average feedback direction. This is consistent with evidence showing that higher uncertainty in predicting aversive events such as pain led to decreased individuals' safety feelings and increased pain perception (reflected by pain ratings and neural correlates) (62). Second, the study by Koban et al. (41) suggests that social feedback information regarding pain works differently from a conditioned learning cue. Although both cause pain ratings to divert toward the predicted cue pain intensity, social information influences appear more robust (higher pain rating and skin conductance response) and involve a different neural network. In addition to social information about pain, even stimulus-independent social information directed to the participants' coping performance (through social comments) has been shown to influence the individual's pain experience (42). This shows how social information received from others—whether specific or non-specific to pain- impacts the individual pain experience. In summary, pain ratings were modulated in all the social feedback studies in this review. The neuroimaging results show that social feedback manipulations primarily influence brain areas related to the affective processing of pain (AI, ACC) and are mediated mainly by the DLPFC, a region associated with cognitive control and pain modulation (63, 64). From the results by Koban et al. (41), in which the two sensory-related neural pain signatures (NPS and SIIPS) were not affected by the modulation, it is plausible to suggest that social feedback influences pain through its affective features. For future studies, it would be essential to test whether one can capture such dissociation at the behavioral level by comparing pain intensity and unpleasantness ratings.

Interestingly, social modulations by the VMPFC were seen only in the study using comments directed at the participant (42) with empathetic comments leading to decreased pain intensity ratings and increased functional connectivity between the VMPFC and anterior and posterior parts of the insula. In that sense, it is reasonable to view the two sub-themes as active vs. passive social information, which might explain such neural difference: social information that is obtained passively (i.e., through observing others' pain ratings) integrates neural processes associated with the attention network and cognitive control [as shown in (41)], while active reception of information by hearing live comments, which are directed at the participant may require further processing related to encoding and integrating of social information, which was shown to recruit the VMPFC (65). Specifically, the pain reduction observed following empathetic comments during pain could also be regarded as a form of social support from the experimenter (who is not a total stranger and has a sense of authority) and, therefore, recruits the VMPFC as shown in the studies of the social support theme. Lastly, some specific effects were also discovered. The study by Yoshida et al. (40) suggests that the PAG encodes the observed uncertainty information from others, leading to uncertainty-induced hyperalgesia. The PAG has been extensively acknowledged for its role in pain modulation (66, 67), which seems to extend to situations with social feedback.

Finally, examining the *group membership* theme studies revealed that manipulations involving entering, exiting, or evaluating group membership concerning an individual can modulate pain ratings. The evidence consistently suggests that being excluded from a group lead individuals to feel negative emotions associated with the experience of rejection, which is followed by an overall increase in pain perception (seen as higher pain ratings during exclusion conditions) (43–45). In turn, including an individual in a group appears to have an analgesic effect (seen by the reduction of pain ratings and increased positive feelings) (43–45). These results are consistent with previous behavioral findings on social rejection and pain (68). Nonetheless, several unique insights can be extracted from this theme. Using a new paradigm, Landa et al. (45) demonstrated that when individuals revisit an inclusive social situation after an experience of being rejected, the hyperalgesic effect could persist depending on whether they still perceived the experience as rejecting (45). Another core new insight regarding group membership comes from the study by Schwarz et al. (46), which showed how stereotypes could direct individuals' pain perceptions and processing according to specific primers. Hence, these findings suggest that group-membership effects can be directional based on a learned primer with a beneficial context (learned from the associated group) or not (45). These findings could open an array of clinical treatments. The patients are assigned/told that their profile/condition is part of another group that exhibits a particular recovery/clinical outcome following treatment.

Interestingly, the finding by Hein et al. (47) that treatment by an outer group member reduced pain (both pain ratings and pain-related neural activation) seems counterintuitive and contradicts those of the studies on social exclusion (i.e., exclusion increases pain while inclusion reduces pain) and social support (support from a close person reduces pain). However, this result might be explained by two critical feature differences—purpose and learned outcome-membership association: In social exclusion, individuals are presented with a particular social interaction with unfamiliar others to which they would prefer to belong (rather than being excluded from the group). In social support, individuals' sense of belongingness to their partner is already grounded. Therefore, in those two themes, the purpose is either to belong (in social exclusion) or to have a sense of belonging (in social support) to others. Whereas, in the in/out-group membership studies, the purpose of the individual is to decide whether/how many others belong to their group. In addition, in the social support and exclusion manipulations, there is no direct control of the other person on participants' pain stimulus *per se*, whereas, in the in/out-group manipulation, the other person directly affects participants' pain. Therefore, prior expectations about the other should be learned and updated if the outcome is wrong (generating a prediction error). In both in/out-group membership and the social exclusion studies, the individual's beliefs about themselves and others shape and modulate pain. In the in/out-group manipulation, beliefs about others help form (and update) a person-outcome association by learning. In the group inclusion/exclusion studies, self-related beliefs affect the degree of perceived exclusion from a group (regardless of whether the individual is excluded). In contrast to the social support and social feedback studies reviewed here, the neuroimaging data show that group-membership manipulations affect sensory (MCC, PI, and thalamus) and affective (ACC and AI) pain-related brain areas. In addition, the amygdala was activated in all the social exclusion manipulations (30, 31, 48), and its activation was also found to be positively correlated with increased pain ratings (43, 44). As the amygdala was previously shown to be involved mainly in the processing of negative emotions (69) as well as in pain modulation (70), it is plausible to suggest its conjoined role with the AI to mediate between the elevated rejection-related emotions (during social exclusion) and their hyperalgesic effects on pain. In the study done by Schwarz et al. (46), the NAc activation was found to decrease with a stereotype associated with decreased pain sensitivity (MLPS vs. FLPS) and to correlate with decreased pain ratings.

Based on previous studies that linked activation changes in the dopaminergic system to stress in which dopaminergic inputs from the ventral tegmental area were shown to be modulated by glutamatergic projections from the amygdala (71–73), activation in the NAc might reflect attenuation of a stress-related signal (during the pain-reducing stereotype).

Finally, it seems that, differently from the other social themes, the primary modulating brain areas of group membership effects on pain are areas of the limbic system (AI, amygdala) rather than prefrontal brain areas such as the VMPFC and DLPFC. Taken together, the yearning to belong or be accepted by a group seems to influence the individual's experience of pain significantly. Even without needing a shared experience with others (like in social feedback), group belonging has a unique and independent effect that adds/subtracts from the negative pain experience by altering mood more generally due to social validation. Group-membership effects seem not to be grounded on others solely but rather a combination of self and other actions/impressions.

### Prefrontal involvement in the modulation of social signals on pain

Next, synthesizing the results of the neural activity during the pain epochs, correlations with pain ratings, and the mediation analyses allow us to infer the role of different prefrontal brain regions and check which are the key players (showing a consistent function) involved in the influence of social cues on pain perception.

### VMPFC

Overall, the social manipulations within the themes of helping others, social support, and social feedback reduced pain ratings. This pain reduction was mostly accompanied by increased neural activity in the VMPFC (30, 34, 37) [but not in (38)] or increased functional connectivity between the VMPFC and pain-related brain areas (29, 42). Reversely, social rejection increased pain ratings, which was accompanied by increased neural activity in the VMFPC (44). These findings imply a selective modulation of pain by the VMPFC, which depends on the valence of the social cue.

Meta-analyses of prefrontal neuroimaging data (21, 74–76) outline that the VMPFC is involved in encoding and representing conceptual information relevant for survival (for the present and the prospective individual's physical and social wellbeing) from environmental and internal cues and in transducing this information into affective behavioral and physiological responses. To generate affective meaning and coordinate emotional behavior, the VMPFC functions as a hub that links systems involved in episodic and semantic memory (77, 78), emotion (79) and emotion regulation (80, 81), social cognition (82, 83), interoceptive signals (80), and subjective values (84).

The role of the VMFPC to modulate pain across different social situations, as identified in this review, fits the suggested function of being a critical hub that integrates different internal and external inputs (visceral, sensory, social) to conceive the meaning of a specific social scenario in order to direct the appropriate behavior/action. Such behavior might be to withdraw from an unpleasant social situation/from others (after social exclusion) or to stay and appreciate the bond with others (e.g., during social support).

### DLPFC

The relationship between neural activity in the DLPFC and pain ratings was positive in the social feedback manipulations (40, 41) and inconsistent in social support (33, 38) and the egocentric interpersonal perception manipulation (32).

As the results are inconsistent within some of the themes, it is only possible to draw general conclusions on the underlying processing of the DLPFC within the reviewed studies. The observed recruitment of the DLPFC might reflect different pain-related processes compared to previous research and might be more prominent in certain themes. These include pain detection (85, 86), pain sensitivity encoding (87), integration of incoming nociceptive signals with cue-based expectation (20), and cognitive control of pain (88). In the reviewed studies, DLPFC involvement could reflect processing related to nociceptive integration, pain detection, and controlling the perceived pain.

### DMPFC

The recruitment of the DMPFC was found in studies employing social manipulations of social support (35, 38) and social conformity (41). From the mediation analyses (41), the positive correlation with pain ratings (38), and the outcome of social support on pain ratings following a DMPFC-iTBS procedure (35), the DMPFC seems to be involved in the encoding of the pain and its modulation during a social situation.

Based on recent meta-analyses on the role of the prefrontal cortex (21, 74), the DMPFC in those themes may be involved in processes related to the appraisal of others' mental states concerning one's well-being (mentalizing and reflection on the self and others) (74), emotion regulation, encoding representation of negative emotions, and general representation of pain (21).

### OFC

In the case of the OFC, the results revealed inconsistent patterns (30, 33, 38), suggesting that the OFC involvement might be exerted indirectly (by influencing other PFC regions) in a pattern that depends on task-specific features/processes.

It appears that the OFC is recruited when an individual is giving (30) or receiving (33, 38) support to/from others. However, as the inconsistent activation pattern also occurs within the social themes, it is difficult to conclude the specific processes within each social theme.

From meta-analytic data of the prefrontal cortex (74, 76), it is plausible to assume that the observed neural activity in the OFC reflects the processing of internal states such as affect and motivation (e.g., when deciding whether to offer help) (76). In addition, it might reflect processes related to goal-directed behavior (giving or preparing to receive help), which include encoding value-outcome associations, and appraisal of episodic memories and imagined future events (anticipated pleasantness of imagined future scenario, real and imagined rewards, imagined future emotional events, and pleasantness and autobiographical memories) (74).

### Critical remarks and suggestions for future research

In this review, several potential issues were noted that would be beneficial to be considered in future studies. The most critical issue was the selection bias of female over male participants (either women only or a highly skewed ratio). Although one could justify such selection by having a more gender-homogenous participant sample, the conclusions of such studies are limited if gender selection is not controlled, matched, and tested for differences. This issue is particularly critical as one study of this review that sampled both genders found a significant difference in social modulation and neural activation during pain (37). In that matter, including more gender identities could be significant and exciting to investigate in future studies on social effects on pain. Many results point to modulations grounded on self-perception that interacts with a particular social situation.

Another critical issue concerns the lack of necessary control conditions: several studies only compared the main manipulation with a contrasting condition without including a control condition independent of the investigated social context. This issue could significantly impact the interpretation of some reported results (e.g., whole-brain neural activations).

A few studies also lacked full/partial details on whole-brain activations (e.g., missing activation tables for each examined condition) and offered brief, vague, or insufficient written descriptions or provided only selected images.

From the pain assessment perspective, there is an imbalance in the usage of the core pain rating scales. While three studies measured pain intensity and unpleasantness, most of the studies measured only intensity (14/19) or unpleasantness (2/19). Indeed, including two sets of rating scales during an experimental task can significantly increase the duration of an experiment. This can be particularly problematic in neuroimaging studies because of the necessity of trial repetition, leading to more subject fatigue, loss of attention, and limiting the inclusion of other conditions in the experiment.

While in most of the studies reviewed here, only pain intensity was measured, several studies included other measures not related to pain (emotion or mood ratings often as a one-time question at the end of the experiment) to provide some insight into the affective-motivational aspect of the painful experience. However, as previous research has shown that perceived pain intensity and unpleasantness are associated with distinct and shared neural representations in the brain (18, 89–92), it should be a consideration in most pain studies to include intensity as well as affective pain scales as outcome measures. This seems especially critical for studies of supraspinal pain modulation.

In the social support theme, there is a large variability in the selection and/or definition of a “romantic partner” by the relationship duration. Therefore, developing a more logical consensus that could be compared across studies is recommended. In addition, a large sample could be tested and used as a covariate or correlational measure in the analyses. Furthermore, it would be essential to compare social support conditions with a neutral condition (“Stranger support”) and to compare negative and positive forms of social support from the same source of support. Allowing to explore the full spectrum of social manipulation might potentially answer whether and when exerting one form of support can have an opposite effect (e.g., viewing a negative facial expression from a romantic partner might still show a positive effect on pain or a positive expression from a stranger might still have a negative effect on pain).

Finally, future studies are encouraged to include chronic pain patients and compare them to healthy populations. Such inclusion might provide critical information for health care providers and clinicians to assess the effectiveness and efficacy of different socially-oriented treatment programs (93–95).

### Summary and conclusion

This review presents and discusses the results of 19 neuroimaging studies examining how social signals influence the individual's experience of pain (see Figure 2 for a summary sketch). By classifying the studies into thematic groups, intra- and inter-thematic mechanisms were discussed and shared, in which distinct modulating factors were identified. As previously theorized by psychosocial pain models, social manipulations robustly influence pain at the level of behavior and neural processing. The final modulatory effect of most social manipulations seems to be dependent on social traits grounded within the self (e.g., perceived helpfulness, perceived rejection, perceived relationship quality, uncertainty sensitivity) and mediated mainly by prefrontal regions (e.g., VMPFC, DLPFC) and brain areas associated with affective processing of pain—mainly the anterior insular cortex. This review adds essential information about neural and behavioral mechanisms to previous reviews on a single thematic topic (14, 16, 17). Hopefully, this review provides a broader perspective and stimulating suggestions for researchers and clinicians.

## Author contributions

GS wrote the manuscript. PS edited it and had manuscript oversight. All authors contributed to the article and approved the submitted version.

## Funding

GS received a salary from a Swiss National Science Foundation (SNSF) project grant to PS (Grant number 320030_179191/1).

## Conflict of interest

The authors declare that the research was conducted in the absence of any commercial or financial relationships that could be construed as a potential conflict of interest.

## Publisher's note

All claims expressed in this article are solely those of the authors and do not necessarily represent those of their affiliated organizations, or those of the publisher, the editors and the reviewers. Any product that may be evaluated in this article, or claim that may be made by its manufacturer, is not guaranteed or endorsed by the publisher.

## Figures and Tables

**Table:** 

**Abbreviation**	**Term**
∞	Correlate
↑	Increase
↓	Decrease
aal3v1	Automated Anatomic Labeling
ACC	Anterior cingulate cortex
adACC	anterior-dorsal ACC
AI	Anterior insula
Amyg	Amygdala
bil	Bilateral
BPD	Borderline personality disorder
CTRL	Control
d	Dorsal
dACC	Dorsal anterior cingulate cortex
DLPFC	Dorsolateral prefrontal cortex
DMN	Default mode network
DMPFC	Dorsomedial prefrontal cortex
EA	Electroacupuncture
EEG	Electroencephalogram
EP	Evoked potential
F	Female
FC	Functional connectivity
fMRI	Functional magnetic resonance imaging
HC	Healthy control
IASP	International Association for the Study of Pain
INS	The whole insular cortex
IPL	Inferior parietal lobe
iTBS	Intermittent theta burst stimulation
l	Left
LEP	Laser evoked potential
LEPs	Laser evoked potentials
LPFC	Lateral prefrontal cortex
M	Male
MCC	Mid cingulate cortex
MEG	Magnetoencephalography
MeSH	Medical subject headings
MFG	Middle frontal gyrus
MI	Mid insula
MNI	Montreal Neurological Institute
MPFC	Medial prefrontal cortex
MTG	Middle temporal gyrus
N.A.	Not applicable. Result or Analysis was not provided.
N1/N100	early (negative) pain evoked potential
N2/N200	late (negative) pain evoked potential
NAc	Nucleus accumbens
NPS	Neurologic Pain Signature
OFC	Orbitofrontal cortex
OMT	Opioid maintenance treatment
P1/P100	early (positive) pain evoked potential
P2/P200	late (positive) pain evoked potential
PAG	Periaqueductal gray
PCC	Posterior cingulate cortex
PET	Positron emission tomography
pgACC	pregenual ACC
PI	Posterior insula
PPC	Posterior parietal cortex
r	Right
Ref.	Reference
ROIs	regions of interests
SCR	Skin conductance response
SFG	Superior frontal gyrus
sgACC	subgenual ACC
SI	Primary somatosensory cortex
SIIPS	Stimulus intensity independent pain signature
SMG	Supramarginal gyrus
STG	Superior temporal gyrus
STS	Superior Temporal Sulcus
TPJ	Temporoparietal junction
VLPFC	Ventrolateral prefrontal cortex
VMPFC	Ventromedial prefrontal cortex
