# Supplementary material for: The influence of social signals on the self-experience of pain: A neuroimaging review
Source: Front Neurol. 2022 Aug 26;13:856874. doi: 10.3389/fneur.2022.856874 (PMC9459049; doi:10.3389/fneur.2022.856874)
Supplement: Supplementary file 1 [file Data_Sheet_1.docx]

# Supplementary Material

## Supplementary Methods – search strategy

### PubMed

#### Relevant MeSH terms

##### Social-related terms

- Behavior and Behavior Mechanisms/
  - Psychosocial Functioning
  - Motivation/
    - Power, Psychological
  - Behavior/
    - **Social Behavior**
    - Information Seeking Behavior
    - Health Behavior
    - Human-Animal Interaction
    - Illness Behavior
    - **Communication/**
      - Cell Phone Use
      - Information Seeking Behavior
      - Nonverbal Communication
      - Verbal Behavior
  - Psychology, Social/
    - Double Bind Interaction
    - **Expressed Emotion**
    - Psychosocial Deprivation
    - **Social Norms**
    - **Social Values**
    - Internal-External Control
    - **Interpersonal Relations/**
      - Disclosure
      - Interprofessional Relations
      - Social Integration
      - Social Interaction
    - **Group Processes/**
      - Consensus
      - Group Structure
      - **Peer Group/**
        - Peer Influence
      - Psychological Distance
      - Role
      - Sensitivity Training Groups
- **Sociological Factors/**
  - Social Change
  - Social Conditions
  - **Social Environment/**
    - Community Networks
    - Social Support
  - Social Isolation
  - Social Norms
  - Socialization
  - Socioeconomic Factors/
    - Social Class
    - Social Factors
- Information Science/
  - **Communication/**
    - Blogging
    - Disclosure
    - **Health Communication**
    - Information Seeking Behavior
    - **Nonverbal Communication**
    - Persuasive Communication
    - **Social Networking**
  - **Communications Media/**
    - Blogging
    - Mass Media
    - Social Media
  - **Internet/**
    - **Internet-Based Intervention**
    - **Internet Use**
    - **Social Media**

##### Therapy / Intervention

- Psychotherapy/
  - **Psychosocial Intervention**
  - Interpersonal Psychotherapy
  - Socioenvironmental Therapy/
    - Psychotherapy, Group/
- Behavioral Disciplines and Activities/
  - **Behavior Control**
- **Internet-Based Intervention**

##### Perception

- Perception/
  - Pain Perception/
    - Nociception
  - Social Perception/
    - Social Cognition

##### Pain

- **Pain/**
  - Acute Pain
  - Chronic Pain
  - Nociceptive Pain
  - Back Pain

##### Methods

- **Pain Measurement**
- **Neuroimaging/**
  - Brain Cortical Thickness
  - Diffusion Tensor Imaging
  - Functional Neuroimaging
- **Tomography/**
  - Magnetic Resonance Imaging
  - Positron-Emission Tomography
- Random Allocation
- Control Groups

##### Coping

- Pain Management

##### Other terms

- Domains & Types
  - Medical Sociology
  - Sociology, Medical
  - Behavioral Medicine
  - Behavioral Research
  - Psychology, Experimental
  - Economics, Behavioral
  - Cognitive Science
  - Psychology
  - Mind-Body Therapies
  - Self-Efficacy
  - **Effect Modifier, Epidemiologic**
  - **Clinical Study**
- Behavior
  - Social Comparison
  - Self-Disclosure
  - Behavior Control

#### Exclusion terms

- Rejection / Social rejection
- Social pain
- Empathy
- Empathy for pain
- Pain, Procedural

#### Search Runs

##### S1 - Search in Title/Abstract “Pain”, “Clinical Pain” and “Social”

(clinical pain[Title/Abstract]) AND (social[Title/Abstract])

Results: 60 studies.

##### S2 - Free search with automatic PubMed labeling

(((("pain"[MeSH Terms] OR "pain"[All Fields]) AND ("social behavior"[MeSH Terms] OR ("social"[All Fields] AND "behavior"[All Fields]) OR "social behavior"[All Fields] OR "sociality"[All Fields] OR "social"[All Fields] OR "socialisation"[All Fields] OR "socialization"[MeSH Terms] OR "socialization"[All Fields] OR "socialise"[All Fields] OR "socialised"[All Fields] OR "socialising"[All Fields] OR "socialities"[All Fields] OR "socializations"[All Fields] OR "socialize"[All Fields] OR "socialized"[All Fields] OR "socializers"[All Fields] OR "socializes"[All Fields] OR "socializing"[All Fields] OR "socially"[All Fields] OR "socials"[All Fields]) AND ("neuroimage"[All Fields] OR "neuroimaged"[All Fields] OR "neuroimagers"[All Fields] OR "neuroimages"[All Fields] OR "neuroimaging"[MeSH Terms] OR "neuroimaging"[All Fields] OR "neuroimagings"[All Fields])) NOT ("empathy"[MeSH Terms] OR "empathy"[All Fields])) NOT ("reject"[All Fields] OR "rejected"[All Fields] OR "rejecter"[All Fields] OR "rejecters"[All Fields] OR "rejecting"[All Fields] OR "rejection, psychology"[MeSH Terms] OR ("rejection"[All Fields] AND "psychology"[All Fields]) OR "psychology rejection"[All Fields] OR "rejection"[All Fields] OR "rejections"[All Fields] OR "rejective"[All Fields] OR "rejects"[All Fields])) AND "journal article"[Publication Type] AND "social behavior"[MeSH Terms] AND (english[Filter])

Results: 56 studies.

##### S3 - MeSH based combinations

- Step 1:
  - (((((("pain"[MeSH Major Topic] AND ("neuroimaging"[MeSH Terms] OR ("positron emission tomography"[MeSH Terms] OR "magnetic resonance imaging"[MeSH Terms])) AND ("social behavior"[MeSH Terms] OR "communication"[MeSH Terms] OR "psychology, social"[MeSH Terms] OR "sociological factors"[MeSH Terms] OR "communications media"[MeSH Terms] OR "internet"[MeSH Terms] OR "psychosocial intervention"[MeSH Terms] OR "behavior control"[MeSH Terms] OR "internet based intervention"[MeSH Terms] OR "social perception"[MeSH Terms])) NOT "social pain"[Title/Abstract]) NOT "social rejection"[Title/Abstract]) NOT "empathy"[MeSH Terms]) NOT "pain, procedural"[MeSH Terms]) NOT "psychological distance"[MeSH Terms]) NOT "injections, spinal"[MeSH Terms]
- Step 2:
  - #Step 1 AND filters
  - (((((((("pain"[MeSH Major Topic] AND ("neuroimaging"[MeSH Terms] OR ("positron emission tomography"[MeSH Terms] OR "magnetic resonance imaging"[MeSH Terms])) AND ("social behavior"[MeSH Terms] OR "communication"[MeSH Terms] OR "psychology, social"[MeSH Terms] OR "sociological factors"[MeSH Terms] OR "communications media"[MeSH Terms] OR "internet"[MeSH Terms] OR "psychosocial intervention"[MeSH Terms] OR "behavior control"[MeSH Terms] OR "internet based intervention"[MeSH Terms] OR "social perception"[MeSH Terms])) NOT "social pain"[Title/Abstract]) NOT "social rejection"[Title/Abstract]) NOT "empathy"[MeSH Terms]) NOT "pain, procedural"[MeSH Terms]) NOT "psychological distance"[MeSH Terms]) NOT "injections, spinal"[MeSH Terms]) NOT "review"[Publication Type]) AND (("clinical trial"[Publication Type] OR "journal article"[Publication Type] OR "randomized controlled trial"[Publication Type]) AND "loattrfull text"[Filter] AND "humans"[MeSH Terms] AND "english"[Language])

Results: 123 studies.

### SCOPUS

#### Search Runs

##### S1 – Based on relevant terms

TITLE ( pain* ) AND TITLE-ABS-KEY ( social* ) AND ( KEY ( neuroimaging ) OR KEY ( fmri ) OR KEY ( functional AND magnetic AND resonance AND imaging ) )

Results: 230 studies.

### Google Scholar

#### Search Runs

##### S1 – Based on relevant terms

allintitle: pain social neuroimaging OR fMRI OR "magnetic resonance imaging" OR "positron emission tomography" OR "PET" OR Neural OR Neuro OR brain OR "brain activity" -"social pain"

Results: 33 studies.

### Total

PubMed (total): 60 + 56 + 123 = 239 studies

Scopus (total): 230 studies

Google Scholar (total): 33 studies.

All sources: 239 + 230 + 33 = 502

Following study selection inclusion/exclusion criteria and removal of duplicates: 19 studies

502

## Supplementary Results

### Supplementary Table 1

|  | *Regions affected by social manipulation during pain  [number of occurrences within/across themes]* | | | | | | |
| --- | --- | --- | --- | --- | --- | --- | --- |
|  | *Within-themes* | | | | | *Across all themes  (15 fMRI studies)* | |
|  | ***Helping others [2]*** | ***Ego. Inter. Perc.  [1]*** | ***Social Support [4]*** | ***Social feedback [3]*** | ***Group membership [5]*** | ***N*** | ***%*** |
| **ACC** | 1 | 0 | 3 | 2 | 3 | 9 | 60% |
| **MCC** | 0 | 0 | 1 | 0 | 2 | 3 | 20% |
| **PI** | 1 | 0 | 1 | 0 | 2 | 4 | 27% |
| **MI** | 1 | 0 | 0 | 1 | 0 | 2 | 13% |
| **AI** | 2 | 0 | 3 | 3 | 4 | 12 | 80% |
| **Thalamus** | 2 | 0 | 3 | 1 | 4 | 10 | 67% |
| **MPFC** | 0 | 1 | 1 | 0 | 0 | 2 | 13% |
| **VMPFC** | 1 | 0 | 2 | 1 | 1 | 5 | 33% |
| **VLPFC** | 0 | 1 | 0 | 0 | 0 | 1 | 7% |
| **DLPFC** | 0 | 1 | 2 | 3 | 0 | 6 | 40% |
| **DMPFC** | 0 | 0 | 0 | 0 | 0 | 0 | 0% |
| **OFC** | 1 | 0 | 2 | 0 | 1 | 4 | 27% |
| **Amyg** | 0 | 0 | 3 | 1 | 2 | 6 | 40% |
| **VTA / NAc** | 0 | 0 | 0 | 0 | 1 | 1 | 7% |
| **Caudate** | 1 | 0 | 1 | 0 | 1 | 3 | 20% |
| **MFG** | 1 | 0 | 1 | 1 | 1 | 4 | 27% |
| **MTG** | 0 | 0 | 0 | 0 | 2 | 2 | 13% |
| **PCC** | 0 | 0 | 0 | 1 | 1 | 2 | 13% |
| **Precuneus** | 0 | 1 | 1 | 1 | 2 | 5 | 33% |
| **IPL/IPS** | 1 | 1 | 0 | 1 | 1 | 4 | 27% |
| **TPJ** | 0 | 1 | 0 | 0 | 0 | 1 | 7% |
| **Pons** | 0 | 0 | 0 | 0 | 1 | 1 | 7% |
| **PPC** | 0 | 0 | 0 | 1 | 0 | 1 | 7% |
| **PAG** | 0 | 0 | 1 | 1 | 0 | 2 | 13% |
| **PCG / SI** | 1 | 0 | 2 | 0 | 2 | 5 | 33% |

### Supplementary Table 2

|  | *Regions correlated with changes in pain ratings  [number of occurrences within/across themes]* | | | | | |
| --- | --- | --- | --- | --- | --- | --- |
|  | *Within-themes* | | | | | *Across all themes  (fMRI studies reporting the correlation)* |
|  | ***Helping others [2]*** | ***Ego. Inter. Perc.  [1]*** | ***Social Support [4]*** | ***Social feedback [3]*** | ***Group membership [5]*** | ***N*** |
| **ACC** | 0 | 0 | 3 | 1 | 0 | 4 |
| **MCC** | 1 | 0 | 0 | 0 | 0 | 1 |
| **PI** | 0 | 0 | 0 | 0 | 0 | 0 |
| **MI** | 0 | 0 | 0 | 0 | 0 | 0 |
| **AI** | 1 | 0 | 2 | 1 | 2 | 6 |
| **Thalamus** | 0 | 0 | 1 | 0 | 0 | 1 |
| **MPFC** | 0 | 0 | 0 | 0 | 0 | 0 |
| **VMPFC** | 0 | 0 | 2 | 0 | 0 | 2 |
| **VLPFC** | 1 | 1 | 1 | 1 | 0 | 4 |
| **DLPFC** | 0 | 0 | 2 | 2 | 0 | 4 |
| **DMPFC** | 0 | 0 | 1 | 1 | 0 | 2 |
| **OFC** | 1 | 0 | 2 | 0 | 0 | 3 |
| **Amyg** | 0 | 0 | 2 | 0 | 2 | 4 |
| **VTA / NAc** | 0 | 0 | 1 | 0 | 1 | 2 |
| **Caudate** | 0 | 0 | 1 | 0 | 0 | 1 |
| **MFG** | 0 | 0 | 0 | 0 | 0 | 0 |
| **MTG** | 0 | 0 | 0 | 0 | 0 | 0 |
| **PCC** | 0 | 0 | 0 | 0 | 0 | 0 |
| **Precuneus** | 0 | 1 | 0 | 0 | 0 | 1 |
| **IPL/IPS** | 0 | 1 | 0 | 1 | 0 | 2 |
| **TPJ** | 0 | 0 | 0 | 0 | 0 | 0 |
| **Pons** | 0 | 0 | 0 | 0 | 0 | 0 |
| **PPC** | 0 | 0 | 0 | 0 | 0 | 0 |
| **PAG** | 0 | 0 | 1 | 1 | 0 | 2 |
| **PCG / SI** | 1 | 0 | 0 | 0 | 0 | 1 |

### Supplementary Table 3

|  | *Psychosocial traits modulating pain ratings/neural activation  [number of occurrences within-themes]* | | | | |
| --- | --- | --- | --- | --- | --- |
|  | *Within-themes* | | | | |
|  | ***Helping others [2]*** | ***Ego. Inter. Perc.  [1]*** | ***Social Support [4]*** | ***Social feedback [3]*** | ***Group membership [5]*** |
| **Positive thoughts** | 1 | 0 | 0 | 0 | 0 |
| **Referencing observed pain** | 0 | 1 | 0 | 0 | 0 |
| **Perceived helpfulness** | 1 | 0 | 0 | 0 | 0 |
| **Relationship quality** | 0 | 1 | 1 | 0 | 0 |
| **Relationship length** | 0 | 0 | 1 | 0 | 0 |
| **Therapeutic alliance** | 0 | 1 | 0 | 0 | 0 |
| **Facial mimicry** | 0 | 1 | 0 | 0 | 0 |
| **Perceived support** | 0 | 0 | 1 | 0 | 0 |
| **Attachment anxiety** | 0 | 0 | 2 | 0 | 0 |
| **Attachment avoidance** | 0 | 0 | 2 | 0 | 0 |
| **Exclusion feeling** | 0 | 0 | 0 | 1 | 0 |
| **Perceived rejection** | 0 | 0 | 0 | 1 | 0 |
| **Personal impression** | 0 | 0 | 0 | 0 | 1 |
